# Associations of History of Displacement, Food Insecurity, and Stress With Maternal-Fetal Health in a Conflict Zone: A Case Study

**DOI:** 10.3389/fpubh.2020.00319

**Published:** 2020-08-13

**Authors:** Doris González-Fernández, Revathi Sahajpal, José E. Chagüendo, Roberth A. Ortiz Martínez, Julián A. Herrera, Marilyn E. Scott, Kristine G. Koski

**Affiliations:** ^1^School of Human Nutrition, McGill University (Macdonald Campus), Sainte-Anne-de-Bellevue, QC, Canada; ^2^Obstetrics and Gynecology Unit, San José Hospital, University of Cauca, Popayán, Colombia; ^3^Department of Family Medicine, School of Medicine, University of Valle, Cali, Colombia; ^4^Institute of Parasitology, McGill University (Macdonald Campus), Sainte-Anne-de-Bellevue, QC, Canada

**Keywords:** symphysis-fundal height, sonography-estimated fetal weight, amniotic-fluid index, internal displacement, stress scale

## Abstract

**Background:** In populations with a history of conflict, early identification of pregnant women who are at risk of adverse pregnancy outcomes is challenging, especially if sonography is not available. We evaluated the performance of symphysis-fundal height (SFH) for identification of high-risk pregnancies and investigated if food security and diet quality, clinical biomarkers, and stress were associated with SFH and two known indicators of maternal-fetal well-being, sonography-estimated fetal weight and amniotic fluid index (AFI).

**Methods:** For this cross-sectional study, 61 women with high-risk pregnancies were recruited after referral to the obstetrics and gynecology unit at San José Hospital in Popayán, Colombia. Multiple stepwise linear and ordered logistic regressions were used to identify associations of SFH, sonography-estimated fetal weight and AFI classification with history of displacement, food insecurity, post-traumatic stress symptoms as well as biopsychosocial risk evaluated through the Colombian risk scale.

**Results:** History of displacement was associated with lower SFH Z-scores, but higher hemoglobin, taking iron supplements and a higher diastolic blood pressure were associated with higher SFH Z-scores. SFH was also associated with AFI but not with sonography-estimated fetal weight. Stress indicators were associated with a higher AFI. In contrast family support, an element of the Colombian biopsychosocial risk assessment, was associated with a higher sonography-estimated fetal weight, whereas more hours of sleep/day were associated with lower sonography-estimated fetal weight.

**Conclusion:** SFH was not only associated with biological factors known to affect maternal/fetal health but also with history of displacement, thus validating its use in conflict areas for pregnancy assessment. Associations of biopsychosocial stressors with maternal-fetal outcomes highlight the need for a systematic assessment of stress in pregnant women from conflict zones.

## Introduction

The World Bank reports that ~2 billion people reside in conflict-affected areas (1). Presently, the United Nations recommends a focus on maternal and neonatal health in conflict zones if targets of the Sustainable Development Goals are to be achieved (2). Colombia has been experiencing armed conflict for over three decades (3) with widespread internal displacement (4) of 6 million people because of violence (5). Internal displacement due to conflict poses physical and mental health risks and leads to disruption of protective factors like community and family networks (1). There is also evidence of slower progress in achieving optimal levels of antenatal care in conflict zones (6).

The impact of conflict on pregnancy outcomes is difficult to measure. Obstetric ultrasound is considered the most reliable and objective way to monitor fetal growth and assess gestational age (GA) (7) and has been successfully used in the prediction of fetal distress in developing countries by measuring fetal movements and amniotic fluid index (8). However, its accessibility, feasibility, and the disclosure of the infant sex and health limits its use in some populations (9). In settings where obstetric ultrasound is not available, a common practice is to measure symphysis-fundal height (SFH) (10). Currently, WHO advocates for further research to determine the association of SFH with fetal growth and risk factors for perinatal morbidity particularly in settings where antenatal ultrasound is not available (11). In Latin America, a reference SFH chart, developed by the Latin American Centre of Perinatology and Human Development (CLAP) is the commonly used (12). Recently, new standards for SFH were adopted by the Intergrowth-21 project, a multicenter and multiethnic study of 8 geographically different countries (13) but studies on the application of these new SFH standards in Latin America are lacking.

Since biopsychosocial factors can contribute to adverse pregnancy outcomes (14), the Colombian Ministry of Health has adopted a biopsychosocial score used to screen pregnant women on the basis of their medical conditions, degree of stress and social/family support, in order to better identify those women at high risk of adverse pregnancy outcomes (15). However, the prevalence of pregnancy complications, particularly small-for-gestational-age babies, continues to be higher in Colombian conflict zones compared with the rest of the country (16), given exposure to conflict-related stress and food insecurity beyond the general difficulties of mothers in low-medium income countries.

Therefore, the objective of this study was to explore if food security and diet quality, clinical biomarkers, history of displacement, and markers of stress were associated with three indicators of maternal-fetal well-being: (1) symphysis-fundal height (SFH), (2) sonography-estimated fetal weight, and (3) sonography-calculated amniotic fluid index (AFI). Stress was measured using scores from both the Colombian biopsychosocial risk scale (15) and the adapted conflict-related stress score (17), in order to assess the possibility that our population may be experiencing post-traumatic stress. We also explored the possibility that SFH could be a useful biomarker to detect pregnancies at risk of adverse outcomes in settings with no access to sonography.

## Methods

### Ethics and Study Population

Ethics approval was obtained from the Scientific Research Ethics of San José Hospital in Popayán, Colombia, by endorsement certificate No. 02 dated April 9, 2018. Participants gave written informed consent in accordance with the Declaration of Helsinki, the Belmont Code and the Federal Regulations Code from the US National Institutes of Health, as stated in Colombian Ministry of Health's Act 008430.

Through a purposive sampling process, we aimed to obtain a representative sample of women from urban and rural areas, from different municipalities and with different risk factors for adverse pregnancy outcomes. The research team estimated that such a sample could be achieved by recruiting women identified as having a high-risk pregnancy during consultation over a 1-month time period at the obstetrics and gynecology service which runs 3 times/week at the San José Hospital in Popayán, the main referral center for patients in the subsidized health care system. Pregnant women of any GA with singleton pregnancies attending the out-patient high-risk-pregnancy clinic between April and May 2018 were asked to participate. Mothers were approached by the principal investigator (DGF) while they were waiting for their appointment. Mothers were informed about the aim of the study, the time that the interview would occur, the volunteer nature of their participation and the non-interference with their regular pregnancy follow-up and treatment. They were told that no financial compensation was provided but that, after understanding their dietary habits, individualized nutritional counseling would be provided.

We were able to recruit 61 mothers referred to this specialized follow-up clinic because of a high-risk pregnancy due to one or more of the following: adolescent pregnancy, multiparity, history of preterm delivery, and morbidities such as hypertensive disorders of pregnancy, anemia, gestational diabetes, or auto-immune disorders (hypothyroidism). All participants were interviewed by the principal investigator after their ambulatory clinical evaluation and none had a critical condition. Of the 61 women, 57 were beyond the 16th week of pregnancy and 48 were >22nd week of pregnancy, the minimal GAs for comparison with the INTERGROWTH standards for SFH and fetal weight growth, respectively (13). We were able to obtain delivery information from 57 women and had missing data on hemoglobin (*n* = 2) and hematocrit (*n* = 2). Multiple linear regression models included the STATA complete-case analysis function (18) which allowed us to both maximize the sample size of each final model and confirm the randomness of missing data using Little's chi-squared test (19).

### Maternal Health Evaluation

Information on obstetric history including risk factor antecedents, history of urogenital infections in the current pregnancy, hemoglobin and hematocrit, weight and height measurements and sonography information were obtained from clinical charts on the day of the interview by the principal investigator. Maternal weight-for-height was compared with the Pan-American Health Organization standards for GA to classify women as underweight, normal, or overweight (12).

Treating physicians took systolic (SBP) and diastolic (DBP) blood pressure using calibrated mercury sphygmomanometers and arm-cuffs adjusted to maternal arm circumference, with the mother in sitting position. The average of blood pressure taken in both arms in extended position at the level of the heart was recorded following recommendations of the Colombian Ministry of Health guidelines (20). Using SBP and DBP from clinical files, mean arterial pressure (MAP), a known risk factor for hypertensive disorders of pregnancy, was calculated as DBP + 1/3 (SBP—DBP) (21) and cut-offs from the United Nations' Women's Health and Education Center were used to define elevated MAP (22). Pulse pressure (PP) was calculated as the difference between SBP and DBP and was considered elevated if >68 mmHg and low if <42 mmHg (23). Anemia was defined as hemoglobin <11 g/dl (24) and normal hematocrit was defined as 35–44% during the first, 30–39% during the second, and 28–40% during the third trimester (25).

### Maternal-Fetal Examination

Mothers at the high-risk pregnancy unit had been referred from peripheral health centers, where basic laboratory and sonography studies were performed. Gestational age at consultation was determined using these early ultrasounds when available. If early ultrasound was not available, date of the last menstrual period was used to estimate GA. In case of inconsistency between date of last menstrual period with a later ultrasound or unreliable last menstrual period (e.g., irregular menstrual cycles, breastfeeding, or using hormonal contraception), the most recent sonography was used.

SFH was measured by physicians at the obstetrics-gynecology department, with the patient lying flat on a bed with her legs extended, using a flexible, non-elastic standard measuring tape extended from the middle of the upper border of symphysis pubis to the highest point of the uterine fundus. SFH in centimeters measured on the day of interview was recorded from hospital records and later translated into Z-scores and centiles using international INTERGROWTH standards (13).

At the time of the interview, most women had obstetric ultrasound performed by a trained obstetrician following the Colombian Ministry of Health Guidelines (20). Sonography-estimated fetal weight and AFI were obtained from hospital records. Sonography-estimated fetal weights (grams) were translated into Z-scores and centiles using international INTERGROWTH standards (26). AFI values <5 cm were considered as oligohydramnios (27) and those >24 cm were considered as polyhydramnios (28). As AFI varies according to GA, we classified AFI as falling into centiles (2.5, 10th, 50th, or 97.5) for GA as described by Machado et al. (29). We also categorized AFI into its <25th, 25th−75th, and ≥75th centiles. A later review of clinical files in December 2019 allowed us to record GA at delivery and birth weights. If mothers did not deliver at the San José Hospital, delivery information was taken from Cauca's Secretary of Health Database. Information on Apgar scores at 1 and 5 min was recorded. Preterm births (<37 weeks GA) and low birth weights (<2,500 g) (30) were identified.

### Socio-Demographic and Psychosocial Questionnaires

Participants completed the following socio-demographic and diet-related questionnaires: (1) a socio-demographic and household assessment (31), (2) the Colombian household food security scale (32), and (3) a food frequency questionnaire based on national guidelines from the Colombian Institute of Family Welfare (Instituto Colombiano de Bienestar Familiar ICBF) (33).

The Colombian biopsychosocial risk scale, which is part of the national guidelines for pregnancy assessment follow-up, was obtained from hospital records. Among all biologic risk factors assessed by the scale, the following were present in our participants: age <16 years (1 point), >35 years (2 points), first pregnancy (1 point), multiparity (>5 gestations, 2 points), previous cesarean section (1 point) or pelvic surgery (1 point), history (1 point), or current (2 points) gestational hypertension, history (1 point) or current (2 points) gestational diabetes, as well as the presence of autoimmune disease (3 points) or presence of anemia (1 point). Psychosocial risks included the presence of emotional distress, depressive mood, and anxiety symptoms (2 or more “intense” items = 1 point). For family support, mothers were asked if they were satisfied with their family or partner support, and answers were recorded in a scale of 0–3 (15). Values 0–1 were considered as low family support. A total scale ≥3 across all questions was classified as high biopsychosocial risk.

In order to determine whether women had experienced a “history of displacement” in response to conflict over the past 50 years, mothers were asked if they had “ever been in a situation of forced displacement due to the armed conflict.”

Women also answered a stress questionnaire adapted from a previously validated Afghan Symptom Checklist score of locally-relevant symptoms of post-traumatic stress in a conflict zone (17). With the adapted score, we assessed the intensity (0 = none, 1 = occasionally, 2 = sometimes, 3 = often) of seven items: recall of stressful events; dreaming about stressful events or nightmares; presence of symptoms like sweating, shaking, or tachycardia (anxiety-like symptoms) when recalling or affected by stressful situations; lack of interest in daily activities; not being able to feel love toward their loved ones; easily startled; and difficulty sleeping. The average score was used to classify women as having no stress (score = 0), mild (score >0 and ≤1), moderate (score >1 and ≤2) and severe stress (score >2). The duration of sleeping at night was also recorded using a scale of 0 (<3 h), 1 (≥3 and <6h), 2 (≥6 and ≤8 h), and 3 (>8 h).

### Statistical Analyses

All statistical analyses were performed using STATA16 (StataCorp, TX, USA), including correlations of food insecurity with history of displacement and with elements of the adapted conflict-related stress questionnaire, the Colombian biopsychosocial risk score, and with weekly intake of foods.

To evaluate the usefulness of SFH as an indicator of fetal growth, we used two approaches: (1) Spearman's correlations between SFH Z-scores and Z-scores of both sonography-estimated fetal weight and birth weights and (2) a multiple regression model for SFH (cm) with sonography-estimated fetal weight (kg) and AFI (cm) as independent variables, while controlling for maternal weight-for-height classification.

In order to determine if maternal characteristics and environmental factors, supplementation/medication variables, dietary variables, and biological factors (presence of infections, hemoglobin, and hematocrit concentrations) were associated with SFH, sonography estimated fetal weight or AFI, we used a three step process. First, for each binary variable (yes/no) where the condition had ≥10 “yes” observations, mean Z-scores of SFH, and sonography-estimated fetal weight were compared using Student's *T*-tests. Second, we assessed associations of our independent variables with SFH Z-scores and sonography-estimated fetal weight Z-scores using linear regressions and with AFI (<25th, 25–75th, and >75th) using ordered logistic regressions. Third, of the six clusters of independent variables, those with a *P* ≤ 0.10 that were not collinear were tested in a backwards stepwise process for inclusion in a single “core” multiple regression model. Then, using the sixth cluster, each element of the stress score, each element of the biopsychosocial score, the composite stress score, and high biopsychosocial risk were entered separately because of collinearity among these variables. The significance level for removal from the model (*P* < 0.10) allowed us to obtain final models with a maximum of 5 variables. If a continuous dependent variable had non-linearity issues in final models, the variable was transformed into its ordinal equivalent as 1: <25th centile, 2: ≥25th and ≤ 75th centile, and 3: >75th centile. Final models with significant stress elements are reported. Missing data were not imputed and complete case analyses resulted in models with *n* = 49–52. Similar percentile- and bias-corrected confidence intervals of bootstrapping reproduced models were used to confirm the stability and unbiased selection of variables in our linear regression models (34). STATA's margins command was used to estimate marginal effects in our ordered logistic regression models (35). Final regressions were evaluated for collinearity (variance inflation factor <10), stability of coefficients (condition number <30), and linearity of associations (augmented component-plus-residual plots and weighted regression lines).

## Results

### Social and Household Characteristics

Maternal characteristics are summarized in Table 1. Mothers' age was 27 ± 7 (mean ± SD) years, had studied 11 ± 3 years. Among our participants 36.1% came from Popayán, the remainder came from 15 municipalities of Cauca and 78.7% lived in urban areas. Most women had finished high school (24.6%) or had technical or university education (44.3%). In general, all urban women benefitted from treated water (80.3%) and sanitation (88.5%), but women from more remote rural areas obtained water from wells or rural aqueducts. Most women had a stable partner (70.5%), 16.4% were not in stable unions, and 13.1% were single. Most households (72.0%) were food secure, but diet quality according to recommendations was inadequate (Table S1).

**Table 1:** Socio-demographic and diet characteristics.

**Place of provenance**	%	**Food insecurity score**	%
Departmental capital	59.0	No food insecurity	72.1
Municipalities with mixed ethnicities^a^	19.7	Mild food insecurity	9.8
Indigenous municipalities^b^	16.4	Moderate food insecurity	8.2
Black municipalities^c^	4.9	Severe food insecurity	9.8
**Household**		**Food insecurity responses**	%
Rural	21.3	Not having sufficient money to buy food	28
Urban	78.7	An adult eats less due to lack of resources	16
**Access to potable water**	80.3	Decrease of meals due to lack of resources	7
**Education level**		Adult skips meals	5
Primary	11.5	Adult complains of hunger	8
Incomplete secondary	19.7	Adult went to bed hungry	5
Completed secondary	24.6	Bought less food for kids due to lack of resources	8
Technical/university	44.3	Child complains of hunger	3
**Occupation**		Child went to bed hungry	2
House	45.9	**Following diet recommendations**	
Technical/professional	19.7	Eggs everyday	67.2
Student	16.4	Dairy products everyday	45.9
Retailing	9.8	Legumes at least 2 times/week	78.7
Other	8.2	Nuts/avocados at least once/week	42.6
**Marital status**		Viscera once/week	32.8
Single	13.1	Fruits and vegetables with every meal	22.5
Unstable partnership	16.4	Limited processed/can meats	80.3
Stable partnership/cohabitation	70.5	Not eating junk food	75.4
**Monthly income**^d^		Use of vegetable oil for cooking	91.8
<1 minimum salary/month	27.8	**Number of food groups for which compliance was achieved**	
1 minimum salary/month	32.9	1–3	18.0
>1 minimum salary/month	39.3	4–6	55.7
		7–9	26.3

a *Mixed ethnic background refers to mestizo, indigenous and black races*.

*^a^Argelia, Bolívar, El Tambo, Mercaderes, San Sebastián, Timbío*.

b *Cajibío, Coconuco, Inzá, Jambaló, Morales, Piendamó*.

c *Patía, Guapi, Santander de Quilichao*.

d *In 2018, a minimum salary was 781,242 COP, equivalent to about 220 USD/month*.

### Clinical Assessment

Maternal and fetal health indicators are summarized in Table 2. Mothers had no evidence of high SBP, DBP, or PP. However, MAP was elevated in 11.5%. In contrast, a PP < 42 mmHg occurred in 67.2% of mothers. Anemia was not a problem as only 4.9% had hemoglobin values <11 g/dL and none had hemoglobin <10 g/dL. However, hematocrit exceeded normal values for GA in 42.6% of the women. Women were prescribed iron (65.6%), calcium (67.2%), and folic acid (59.0%) supplements, and 41.0% purchased multivitamin-mineral supplements.

**Table 2:** Maternal and fetal health indicators.

**Maternal clinical information**		**Sonography measurements (*n =* 61)**	
Gestational age at interview, median (min-max)	31.4 (16.3–38.6)	Gestational age at sonography (weeks), median (min-max)	31.2 (7.4–38.2)
**Weight-for-height classification (*n =* 61)**		**Fetal weight (grams) in women with GA ≥20 weeks (*n =* 51)**, mean ± SD	1926.4 ± 790.9
Underweight, %	9.8	Fetal weight Z-score, mean ± SD	0.07 ± 0.81
Normal weight, %	42.6	Fetal weight centiles median (min-max)	54.5 (5.4–96.9)
Overweight	47.5	Fetal weight <10th centile, %	5.9
**Blood pressure measurements (mmHg) (*n =* 61)**		**Amniotic fluid index (*n =* 50)**, mean ± SD	13.9 ± 4.1
Systolic blood pressure, mean ± SD	112 ± 12	Centile classification for GA	
Diastolic blood pressure, mean ± SD	68 ± 9	Amniotic fluid index <10th centile, %	14.0
Mean arterial pressure, mean ± SD	83 ± 10	Amniotic fluid index >90th centile, %	4.0
Pulse pressure, mean ± SD	43 ± 8	Low and high AFI in our population	
Elevated mean arterial pressure, %	37.7	Amniotic fluid index <25th centile, %	22.0
Pulse pressure <42 mmHg, %	57.4	Amniotic fluid index >75th centile, %	24.0
**Hematological variables (*n =* 59)**		**Newborn characteristics (*n =* 57)**	
Hemoglobin, g/dL, mean ± SD	12.8 ±1.2	Gestational age at delivery (weeks), median (min-max)	38.5 (29.3–41.0)
Hematocrit, percentage, mean ± SD	38.7 ± 3.8	Preterm delivery (<37 weeks), %	14.0
High Hematocrit, %	42.6	**Birth weight (g)**, mean ± SD	3052.0 ± 492.5
Anemia (hemoglobin <11 g/dL), %	4.9	Birth weight Z-score, mean ± SD	0.04 ± 0.83
**Symphysis-fundal height (SFH, cm) (*n =* 53)**, mean ± SD	27.0 ± 5.1	Birth weight <2,500 g, %	12.3
SFH Z-score, mean ± SD	−1.53 ± 1.16	Apgar score at 1 min, ≥8, %	96.5
SFH centile, median (min-max)	7 (0–85.4)	Apgar score at 5 min, ≥8, %	100.0
SFH <10th centile, %	64.1		

*Elevated mean arterial pressure: >87 mmHg (10–18 week), >84 mmHg (18–34 week), and >86 mmHg (after week 34) (22)*.

*High hematocrit: >41 %(1st trimester), >39 % (2nd trimester), >40% (3rd trimester) (25)*.

### Pregnancy and Offspring Characteristics

**Figure 1:**
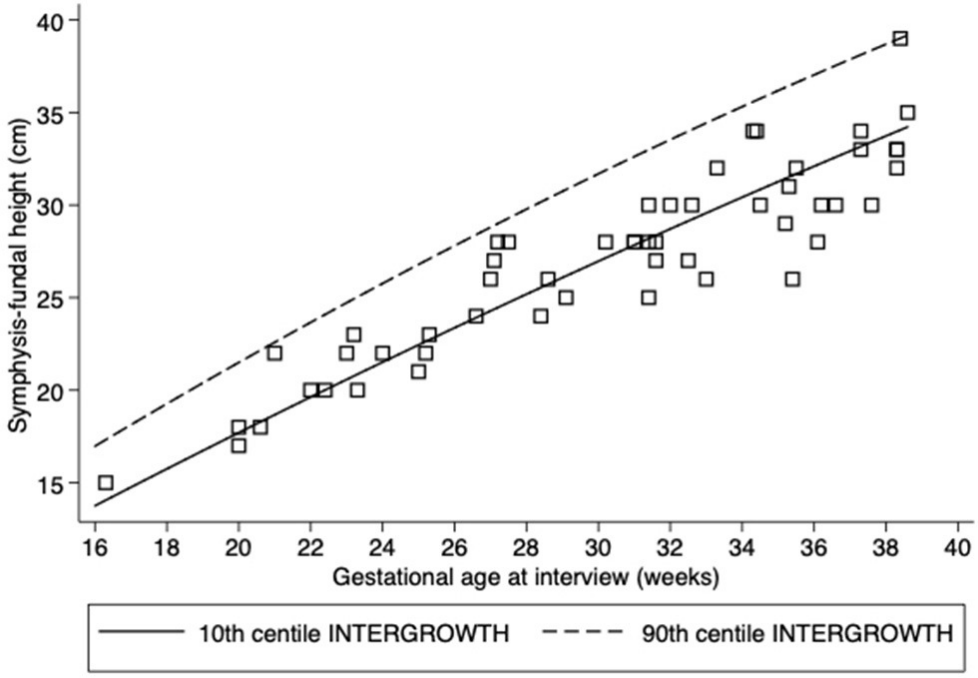
Scatter plots of symphysis-fundal height (cm). Lines correspond to the 10 and 90th centiles according to INTERGROWTH standards.

**Figure 2:**
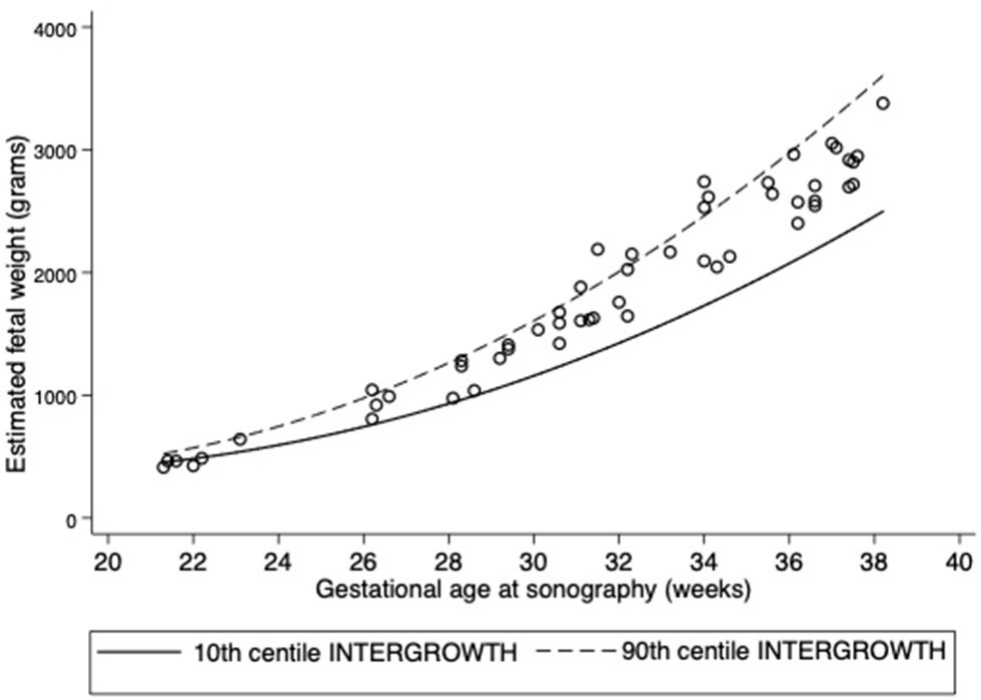
Scatter plots of sonography-estimated fetal weight (grams). Lines correspond to the 10 and 90th centiles according to INTERGROWTH standards.

At interview, 3.3% women were in their first trimester, 18.0% were in their second, and most (78.7%) were in their third trimester. INTERGROWTH standards for SFH and sonography-estimated fetal weight revealed that SFH values were <10th centile in 64.1%, but only 5.9% of sonography-estimated fetal weights were <10th centile (Table 2, Figures 1, 2). A low AFI was found in 14.0% and a high AFI in 4.0% (Table 1).

### Assessment of Maternal Stress

None of the women reported recent displacement, but 29.5% of the mothers reported a history of displacement. However, in the adapted conflict-related stress questionnaire (Table 3A), 8.2% reported no stress, 72.1% had mild stress, 13.1% had moderate stress, and 6.6% reported severe stress in the current pregnancy. Of note, symptoms of both anxiety and depression and also sleep disturbances were common (Table 3A).

**Table 3:** Items and scores for (A) Adapted conflict-related stress and (B) Colombian biopsychosocial risk assessment.

**A. Adapted conflict-related stress questionnaire^**a**^**	**Prevalence %**	**B. Colombian biopsychosocial risk score^**b**^**	**Score**	**Prevalence %**
Recalling stressful events	49.2	**Biological risk factors**		65.6
Dreaming about stressful events	31.1	<16 years	1	3.3
Anxiety-like symptoms	42.6	>35 years	2	13.1
No interest in daily activities	36.1	First pregnancy	1	29.5
Not feeling love toward relatives and friends	31.1	>4 pregnancies	2	6.6
Easily startled	45.9	Anemia	1	4.9
Difficulty in sleeping	54.1	History of pre-term delivery	1	16.4
**Stress score**		History of hypertension	1	13.1
0 = stress	8.2	Current hypertension	2	3.3
>0 and ≤1: mild stress	72.1	History of pelvic surgery	1	24.6
>1 and ≤2: moderate stress	13.1	History of gestational diabetes	2	9.8
>2: severe stress	6.6	Auto-immune disease (hypothyroidism)	3	6.6
**Hours of sleep**		**Psychosocial risk factors**		62.3%
<3 h	4.9	Emotional tension	1 point if severe	36.0
3–6 h	16.4	Depressive mood		24.6
6–8 h	47.5	Neurovegetative symptoms^c^		9.8
>8 h	31.2	**Family support**^**d**^		
		No family support	1 point if absent family support^c^	6.6
		Some family support		3.3
		Moderate family support		4.9
		Strong family support		85.2
		**High biopsychosocial risk**	**Score** **≥3**	24.6

a *Miller et al. (17)*.

b *Herrera (15)*.

c *Hands sweating, mouth dryness, blushing, pallor, tensional headache*.

d *Family support: mothers were asked if they were satisfied with their family or partner support, which was classified as always, sometimes or never. Reporting never resulted in a score of 1 for the general biopsychosocial risk assessment*.

Regarding the Colombian biopsychosocial assessment tool (Table 3B), 65.6% had at least one of the biological risk factors that included adolescent pregnancy, grand multiparity, history of preterm delivery, gestational diabetes, and/or hypertensive disorders of pregnancy, 62.3% reported having psychological risk factors and 9.8% had low or no family support; most women (90.1%) reported moderate or high family support. However, when assessing the overall biopsychosocial risk, 24.6% were at high risk for adverse pregnancy outcomes (score ≥ 3).

### Correlations of History of Displacement and Food Insecurity With Diet and Stress Variables

Importantly, history of displacement was not correlated with elements of the adapted stress questionnaire, the Colombian biopsychosocial risk score or frequency of intake of food groups. On the other hand, food insecurity score was correlated with our stress questionnaires. For the adapted stress questionnaire, higher food insecurity correlated with having no interest in daily activities, not feeling love toward relatives and friends, having difficulty sleeping, recalling stressful events, anxiety-like symptoms, being easily startled, and having difficulty sleeping. For the Colombian biopsychosocial risk scale, higher food insecurity correlated with emotional tension, with depressive mood, with neurovegetative symptoms, and with less family support. With regards to diet quality and compliance with Colombian dietary recommendations, food insecurity was correlated with lower intake of dairy products, nuts/avocados, legumes, fruits, and vegetables (Table S2), and with less hours of sleep.

### Factors Associated With Fetal Growth

Comparison among means of SFH Z-scores by the presence/absence of several maternal characteristics is described in Table 4. Lower SFH Z scores were found in women with a history of displacement and in those taking multivitamins whereas higher SFH Z-scores occurred in women receiving iron or calcium supplements (Table 4A). A similar comparison for sonography-estimated fetal weight showed that Z-scores were significantly lower in women living in rural areas, those with low incomes and those who slept >8 h/day but higher in women who slept <6 h (Table 4B).

**Table 4:** Comparisons of symphysis-fundal height (SFH) Z-scores and sonography-estimated fetal weight Z-scores by binary maternal variables.

	**A. Symphysis-fundal height Z-scores mean** **±** **SD**, ***n*** **=** **53**			**B. Sonography-estimated fetal weight mean** **±** **SD**, ***n*** **=** **51**		
**Condition**	**yes**	**no**	***P***	**yes**	**no**	***P***
History of displacement	−2.1 ± 0.3 (*n =* 15)	−1.3 ± 0.2 (*n =* 38)	0.011	−0.21 ± 0.21 (*n =* 14)	0.17 ± 0.13 (*n =* 37)	0.065
Rural household	−1.71 ± 0.44 (*n =* 10)	−1.48 ± 0.17 (*n =* 43)	0.29	−0.36 ± 0.27 (*n =* 10)	0.17 ± 0.12 (*n =* 41)	0.031
Low income	−1.53 ± 0.23 (*n =* 33)	−1.52 ± 0.20 (*n =* 20)	0.48	−0.07 ± 0.15 (*n =* 33)	0.32 ± 0.14 (*n =* 18)	0.047
Iron supplement	−1.27 ± 0.2 (*n =* 34)	−1.98 ± 0.26 (*n =* 19)	0.016	0.04 ± 0.16 (*n =* 31)	0.11 ± 0.14 (*n =* 20)	0.37
Calcium supplement	−1.30 ± 0.19 (*n =* 35)	−1.95 ± 0.27 (*n* = 18)	0.027	0.03 ± 0.15 (*n =* 32)	0.12 ± 0.15 (*n =* 19)	0.35
Multivitamins	−1.92 ± 0.23 (*n =* 21)	−1.26 ± 0.20 (*n =* 32)	0.021	0.05 ± 0.16 (*n =* 20)	0.08 ± 0.15 (*n =* 31)	0.45
Sleeps >8 h/day	−1.37 ± 0.26 (*n =* 14)	−1.53 ± 0.19 (*n =* 39)	0.33	−0.39 ± 0.22 (*n =* 14)	0.24 ± 0.12 (*n =* 37)	0.005
Sleeps <6 h/day	−1.21 ± 0.45 (*n =* 12)	−1.62 ± 0.16 (*n =* 41)	0.14	0.44 ± 0.22 (*n =* 13)	−0.06 ± 0.13 (*n =* 38)	0.027

#### Symphysis-Fundal Height

SFH Z-scores were significantly correlated with sonography-estimated fetal weight Z-scores (*r*_s_ = 0.34, *P* = 0.017), AFI (*r*_s_ = 0.35, *P* = 0.015), and birth weight Z-scores (*r*_s_ = 0.32, *P* = 0.021), but with low correlation coefficients. However, our multiple linear regression revealed that SFH in cm was positively associated with GA (standardized β-coefficient = 0.89, *P* < 0.0001) and AFI (cm) (β = 0.13, *P* = 0.023) but not with sonography-estimated fetal weight (kg) (β = 0.02, *P* = 0.72) when adjusting for maternal weight-for-height classification (full model *R*^2^ = 0.85 and *P* < 0.0001).

#### Determinants of Symphysis-Fundal Height Z-Scores

The stepwise model for SFH Z-scores captured 26% of the variability and showed that history of displacement was associated with lower SFH Z-score whereas higher MAP was associated with higher SFH Z-scores (Table 5A). In order to observe which component of MAP was associated with SFH, SBP and DBP were included in separate models because of collinearity. Higher DBP, higher hematocrit and taking iron supplements were positively associated with SFH Z-scores. This model captured 38% of variability in SFH Z-score (Table 5B).

**Table 5:** Multiple linear regression for Symphysis-fundal height (SFH) Z-scores, with (A) mean arterial pressure (MAP) and hemoglobin (grams/dL) and (B) diastolic blood pressure (DBP) and hematocrit (%).

**A. SFH Z-score^a^^,^^c^**	**Non-standardized Coef. ± SE**	***P***	**95% CI**	**Standardized β-coef**.	**Overall model**
History of displacement	−1.01 ± 0.31	0.001	−1.72, −0.48	−0.36	*P* = 0.0010 Adj. *R*^2^ = 0.262
*MAP, centile^*b*^*	0.49 ± 0.20	0.017	0.09, 0.89	0.30	
Hemoglobin, grams/dL	0.36 ± 0.21	0.096	−0.07, 0.79	0.21	
Taking iron supplements (0 = no, 1 = yes)	0.54 ± 0.29	0.073	−0.05, 1.39	0.22	
Constant	−3.40 ± 0.62	<0.0001	−4.65, −2.14		
**B. SFH Z-score**^a^, ^c^	**Non-standardized Coef**. **±** **SE**	***P***	**95% CI**	**Standardized** **β-coef**.	**Overall model**
History of displacement	−1.09 ± 0.31	0.001	−1.72, −0.48	−0.41	*P* < 0.0001 Adj. *R*^2^ = 0.379
Taking iron supplements (0 = no, 1 = yes)	0.59 ± 0.28	0.041	0.02, 1.15	0.24	
Hematocrit, centile	0.47 ± 0.18	0.014	0.10, 0.84	0.29	
*Diastolic blood pressure, centile^*b*^*	0.91 ± 0.25	0.001	0.39, 1.43	0.41	
Constant	−4.37 ± 0.65	<0.0001	−5.68, −3.06		

a *Variables that were included but were not retained in the final models (P > 0.10): # sleep hours (score), eating nuts/avocado at least once/week (0 = no, 1 = yes), history of hypertensive disorders of pregnancy (0 = no, 1 = yes)*.

*Model A: n = 52. VIF = 1.02. Condition number: 10.63*.

*Model B: n = 50. VIF = 1.03. Condition number: 12.41*.

b *25th and 75th centiles of MAP, DBP, hemoglobin and hematocrit were calculated from our population and used instead of continuous variables in order correct for non-linear associations: MAP: <73, between 73 and 83 and >83 mmHg, DBP: <60, between 60 and 70 and >70 mmHg; hemoglobin: <12, ≥12 and ≤13.7, >13.7 grams/dL; hematocrit: <35.7, ≥35.7 and ≤41.2, >41.2%*.

c *After 50 bootstrap replications of linear regression models we observed that percentile- and bias-corrected confidence intervals were similar (Table S3)*.

#### Determinants of Sonography-Estimated Fetal Weight-Z-Scores

Our final model for sonography-estimated fetal weight Z-scores, which captured 20.7% of its variability, showed that lower fetal weight was associated with low or no family support and with more hours of sleep (Table 6).

**Table 6:** Multiple linear regression model for sonography-estimated fetal weight Z scores.

**Fetal weight Z–score^**a**^**	**Non–standardized Coef. ± SE**	***P***	**95% CI**	**Standardized β-coef**.	**Overall model**
Hours of sleep category^b^	−0.33 ± 0.13	0.011	−0.59, −0.08	−0.352	*P* = 0.0029 Adj. *R*^2^ = 0.207
*Low or no family support*^c^	−0.75 ± 0.32	0.025	−1.40, −0.10	−0.302	
Weekly intake of legumes (score)^d^	−0.12 ± 0.07	0.097	−0.25, 0.02	−0.231	
Constant	1.21 ± 0.32	<0.0001	0.57, 1.85		

a *Variables that were included but were not retained in the final model (P > 0.10): Not stable union, rural household, aspirin intake (0 = no, 1 = yes), low income, presence of food insecurity (0 = no, 1 = yes). After 50 bootstrap replications of linear regression models we observed that percentile- and bias-corrected confidence intervals were similar (Table S3). n = 51. VIF = 1.12. Condition number: 6.98*.

b *Self-reported hours of sleep were categorized as: 0 if <3 h, 1 if ≥3 h and <6 h, 2 if ≥6 h and ≤8 h, 3 if >8 h*.

c *Low or no family support was categorized as: 0 = moderate or strong, 1 = low or no family support*.

d *Intake of legumes was categorized as: 0 = never, 1 = occasionally, 2 = 1 day/week, 3 = 2–3 days/week, 4 = >3 days/week, 5 = everyday*.

### Amniotic Fluid Index

Multiple ordered logistic regression models for AFI (<25th, 25th−75th, and >75th centile) with elements of the adapted conflict-related stress questionnaire and the Colombian biopsychosocial risk assessment are described in Table 7. Severe lack of interest in daily activities was associated with increased odds of a larger AFI [odds ratio (OR) 8.44, 95% CI: 1.29, 54.9]. A second model for AFI showed that low family support was also associated with an increased odds of a larger AFI (OR: 9.05, 95% CI: 1.18, 69.2) (Figure S1), whereas the intake of folic acid supplements was associated with lower odds (OR: 0.30, 95% CI: 0.09, 0.99) (Table 7).

**Table 7:** Multiple ordered logistic regression models for amniotic fluid index for elements of (A) the adapted conflict-related stress questionnaire and (B) the Colombian biopsychosocial risk assessment^a^.

**A. AFI and interest in daily activities^**b**^**	**OR ± SE**	***P***	**95% CI**	**Overall model**
GA at sonography (week)	0.93 ± 0.06	0.308	0.82, 1.06	*P* = 0.0359 Pseudo *R*^2^ = 0.118
Lack of interest for daily activities (0 = none, 1 = mild, 2 = moderate, 3 = severe)				
Mild	2.28 ± 1.70	0.270	0.53, 9.89	
Moderate	10.59 ± 14.60	0.089	0.70, 159.21	
Severe	8.44 ± 8.07	0.026	1.29, 54.91	
Maternal age (yr)	0.93 ± 0.04	0.087	0.86, 1.01	
**B. AFI and low family support**^**c**^	**OR** **±** **SE**	***P***	**95% CI**	**Overall model**
GA at sonography (week)	0.90 ± 0.06	0.114	0.79, 1.02	*P* = 0.0112Pseudo *R*^2^ = 0.110
Folic acid supplementation (no = 0, yes = 1)	0.30 ± 0.18	0.048	0.09, 0.99	
Low or no family support^d^	9.05 ± 9.39	0.034	1.18, 69.25	

a *AFI, Amniotic fluid index expressed as centiles as follows: AFI <25th centile (<12.3 cm), ≤25th and ≥75th centile (12.3–16 cm) and >75th centile (>16 cm) according to our population's AFI distribution*.

b *Model A: n = 50. VIF = 1.03. Condition number: 12.03. Variables that were included but were not retained in the final model (P > 0.10): maternal age, taking folic acid supplements, decreased number of meals because of lack of resources, eating more eggs/week, sonography-estimated fetal weight*.

c *Model B: n = 50. VIF = 1.01. Condition number: 12.50. Variables that were included but were not retained in the final model (P > 0.10): taking folic acid supplements, decreased number of meals because of lack of resources, eating more eggs/week, sonography-estimated fetal weight*.

d *Low or no family support was categorized as: 0 = moderate or strong, 1 = low or no family support*.

## Discussion

The impact of conflict on pregnancy outcomes is difficult to measure due to its intrusion in all aspects of society including antenatal care. We had the opportunity address associations of conflict-related stress with maternal-fetal outcomes including SFH which, in remote areas, is the only available tool for health professionals. Moreover, given that some women reached a tertiary care center, sonography was also available. This allowed us to explore determinants of pregnancy outcomes for SFH, sonography-estimated fetal weight, and AFI. Several important observations emerged from this case study. First, history of displacement was an independent event that was unrelated to indicators of stress, food insecurity, and diet. Second, lower SFH Z-scores were associated with history of displacement whereas higher MAP or DBP, elevated hematocrit and iron supplementation were associated with higher SFH Z-score. Third, challenging the assumption that SFH is a proxy for fetal growth, our data showed that SFH was significantly associated with amniotic fluid index but not fetal weight. Fourth, two indicators of depression in the current pregnancy, sleeping and severe lack of interest in daily activities, were associated with lower sonography-estimated fetal weight and with higher AFI, respectively. Interestingly, these two conditions were mitigated by evidence of stronger family support. Together these results suggest that food insecurity had an indirect association with maternal and fetal outcomes, whereas different indicators of stress were directly associated with SFH, AFI, and sonography-estimated fetal weight.

### History of Displacement Was Associated With Lower Symphysis-Fundal Height

Although there is evidence of an association between psychosocial stress and lower birthweight in pregnant women in the current pregnancy (36), less is known about the long-term effects of stressful situations in future pregnancies. In our population, 29.5% of the mothers had a history of displacement, which we show for the first time to be associated with lower SFH. It has been reported that symptoms of post-traumatic stress (37) are associated with adverse maternal outcomes including gestational diabetes, preeclampsia (38) and spontaneous preterm birth (39), which, respectively, were 9, 12.3, and 14% in our study population. Although women reported several symptoms included in the adapted conflict stress score, none of them were directly associated with our outcomes. Therefore, our findings would suggest that the impact of conflict-related displacement may persist but this requires further investigation.

### Stress and Sonography Measurements

We found evidence that stress was associated with maternal/fetal outcomes. More severe anxiety-like symptoms and lacking interest in daily activities were associated with increased odds of a larger AFI. Large amniotic fluid volume has been associated with adverse maternal outcomes such as gestational diabetes and pregnancy-induced hypertension (28), as well as with adverse neonatal outcomes including fetal distress (40), macrosomia (41), abnormal presentation, and neonatal death (28). We showed that higher AFI values, though within normal ranges (>16 cm), were associated with maternal psychological symptoms. Thus, AFI in the highest centile may be an overlooked potential indicator of maternal/fetal distress as suggested by La Marca et al. who found an association of chronic social overload with higher concentrations of stress hormones in amniotic fluid (42). Our findings highlight the need to incorporate assessment of stress in order to identify women at risk of adverse pregnancy outcomes.

We also observed that more self-reported hours of sleep were associated with lower fetal weights. Prolonged nocturnal sleep is known to be associated with mood disorders including depression in the general population (43) and also during pregnancy (44, 45). Depression during pregnancy is a risk factor for preterm delivery and SGA (46). Moreover, we observed that lack of interest in daily activities, another symptom of depression (45), was associated with increased odds of a larger AFI. Thus, the possibility of underlying depression, suspected in women who had more hours of sleep and lack of interest in daily activities, is plausible and warrants further investigation.

Interestingly, the presence of low family support was also associated with a higher AFI and with lower sonography-estimated fetal weight. Resilience resources such as social support, personal beliefs and values, education and healthy behavioral practices have been identified as possible mitigators of stress with potential benefits on pregnancy outcomes (47). There is evidence that low social or family support is associated with impaired fetal growth (48) and that intimate social support from a partner or family member improves fetal growth (49). Both are consistent with our findings.

### Indicators of Maternal Health and Well-Being

Among blood pressure measurements, higher MAP has been associated with altered placental perfusion (50) and among supplements, iron given under normal hemoglobin conditions, can lead to hemoconcentration with further increased blood viscosity, oxidative stress, and decreased perfusion of the fetus (51). On the other hand, it has also been demonstrated that an adequate perfusion measured through DBP is necessary for fetal growth (52). Interestingly, the continuous variables for DBP and MAP, as well as iron supplementation were positively associated with SFH Z-score, but were not associated with sonography-estimated fetal weight or AFI. Supporting our findings, there is evidence that lower SFH Z-scores are associated with lower pulse pressure in a vulnerable indigenous population (53). Given the complexity of the SFH measurement, associations of blood pressure measurements with SFH warrant further investigation.

## Strengths and Limitations

We acknowledge three main limitations in our study. First, we recognize that this is a cross-sectional pilot study with a small sample size. However, we were able to recruit >90% of high risk pregnancies referred to the regional hospital; only three did not attend the post-consultation interview for the project.

Second, we recognize that SFH has low sensitivity and specificity for the detection of small-for-gestational age infants, which limited our ability to identify this condition in our study population.

Third, given that we only met with women once it was difficult to explore the sensitive issue of history of displacement in depth.

Despite these limitations, an important strength of this study was the ability to identify for the first time an overlooked association of stress in a conflict zone with clinical indicators of maternal-fetal health including SFH, sonography estimated fetal weight, and AFI.

## Conclusion

In conclusion, history of displacement and elements of the biopsychosocial stress score were associated with several quantitative biomarkers of maternal-fetal health. Our findings collectively point to the need for the integration of assessment of psychological risk factors together with measures of social and family support along with SFH and sonography measurements when identifying high-risk pregnancies to mitigate the impact of conflict-related stresses in vulnerable populations. We provide evidence that SFH may be useful in assessing maternal/fetal health when sonography is not available.

## Data Availability Statement

The datasets generated for this study may be available on written request to the corresponding author.

## Ethics Statement

The studies involving human participants were reviewed and approved by Scientific Research Ethics Board of San José Hospital by endorsement certificate no. 02 from April 9, 2018. The patients/participants provided their written informed consent to participate in this study as stated in Colombian Ministry of Health's Act 008430.

## Author Contributions

DG-F, RS, and KK wrote the initial manuscript. DG-F and RS ran statistical analyses. DG-F was responsible for inter-institutional collaborations, participated in the study design, submitted ethical approvals, interviewed participants, collected information from the files, and created the database. JC and RO, obstetricians, collected clinical data, facilitated inter-institutional collaboration with the University of Cauca, and the process of ethical approval and critically read the paper. JH facilitated interinstitutional collaborations and made important contributions to the biopsychosocial aspects of the manuscript. MS and KK participated in the study design and provided technical advice during field data collection. DG-F and KK contributed to the funding of the project. All authors read and approved the content of the article.

## Conflict of Interest

The authors declare that the research was conducted in the absence of any commercial or financial relationships that could be construed as a potential conflict of interest.
