# Supplementary material for: Associations of History of Displacement, Food Insecurity, and Stress With Maternal-Fetal Health in a Conflict Zone: A Case Study
Source: Front Public Health. 2020 Aug 13;8:319. doi: 10.3389/fpubh.2020.00319 (PMC7438926; doi:10.3389/fpubh.2020.00319)

Supplementary Material

1 Supplementary Figures and Tables

Table S1 Frequency of intake of selected food groups in 61 women from a conflict zone.

A. Weekly intake, n	Everyday	>3 d/week	1-3 d/week	1 time/week	Occasionally	Never
Eggs	41	14	2	2	1	1
Dairy products	28	8	7	4	11	35
Legumes	20	17	11	5	4	4
Nuts/Avocados	8	2	9	7	16	19
Viscera	2	2	8	8	16	25
Processed/can meats	0	3	3	6	12	37
Junk food	4	2	5	4	18	28
**B. Daily intake, n**	**3 times/d**	**2 times/d**		**1 time/d**		**No intake**
Fruits and vegetables	22.9	27.9		34.4		14.7

Table S2 Correlations between the degree of food insecurity and elements of the adapted stress questionnaire, the Colombian biopsychosocial risk score and of the diet with stress variables

**Food insecurity scale and adapted stress questionnaire**		**Food insecurity scale and biopsychosocial risk score**		**Food insecurity scale and weekly intake of foods**	
Recalling stressful events	0.31*	Emotional tension	0.40**	Dairy products	-0.29*
Anxiety-like symptoms	0.29*	Depressive mood	0.44***	Nuts, avocados	-0.26*
No interest in daily activities	0.58****	Neurovegetative symptoms	0.30*	Legumes	-0.29*
Not feeling love towards relatives and friends	0.38**	Family support	-0.27*	Fruits and vegetables	-0.31*
Easily startled	0.30*				
Difficulty in sleeping	0.36**				
**Stress score**	**0.47*** **	**Biopsychosocial risk**	**0.39** **	**Food groups score**	**-0.29* **

* P <0.05, ** P <0.01, *** P <0.001, **** P <0.0001

Table S3 Bootstrap standard error and normal, percentile and bias-estimated confidence intervals using 50 bootstrap replications for models of symphysis-fundal height (SFH) Z-scores, and sonography-estimated fetal weight with an estimate of their bias.

**A. SFH Z-score**	**Observed coefficient**	**Bias**	**Bootstrap SE**	**Bootstrap normal-based CI**	**Bootstrap percentile CI**	**Bootstrap bias-corrected CI**
History of displacement	-0.99	-0.03	0.31	-1.60, -0.38	-1.61, -0.45	-1.53, -0.30
MAP, centile	0.57	-0.02	0.22	0.13, 1.01	0.13, 0.91	0.18, 1.13
Hemoglobin, grams/dL	0.30	0.02	0.11	0.09, 0.52	0.15, 0.54	0.15, 0.54
**B. SFH Z-score**						
History of displacement	-1.15	-0.001	0.32	-1.77, -0.53	-1.73, -0.48	-1.73, -0.48
Diastolic blood pressure, centile	0.91	0.003	0.20	0.52, 1.30	0.52, 1.22	0.52, 1.22
Taking iron supplements	0.61	0.004	0.26	0.09, 1.13	0.19, 1.19	0.20, 1.29
Hematocrit, %	0.09	0.001	0.03	0.03, 0.16	0.03, 0.15	0.04, 0.18
**C. Sonography-estimated fetal weight Z-score**						
Stress score (0-3)	-0.16	0.01	0.19	-0.53, 0.21	-0.52,0.15	-0.58, 0.15
SFH Z-scores	0.26	0.006	0.07	0.12, 0.40	0.13, 0.39	0.07, 0.39
Hours of sleep[5]	-0.36	0.007	0.11	-0.59, -0.14	-0.57, -0.13	-0.57, -0.13
Aspirin intake	-0.48	0.010	0.23	-0.93, -0.02	-0.79, 0.007	-0.79, 0.15

2 Supplementary Figures

Supplementary Figure 1. Predicted probability of amniotic fluid index (AFI) (low-medium-high) by (A) severity of lack of interest in daily activities controlling for gestational age and maternal age and (B) low family support, controlling for gestational age and intake of folic acid supplements.

A

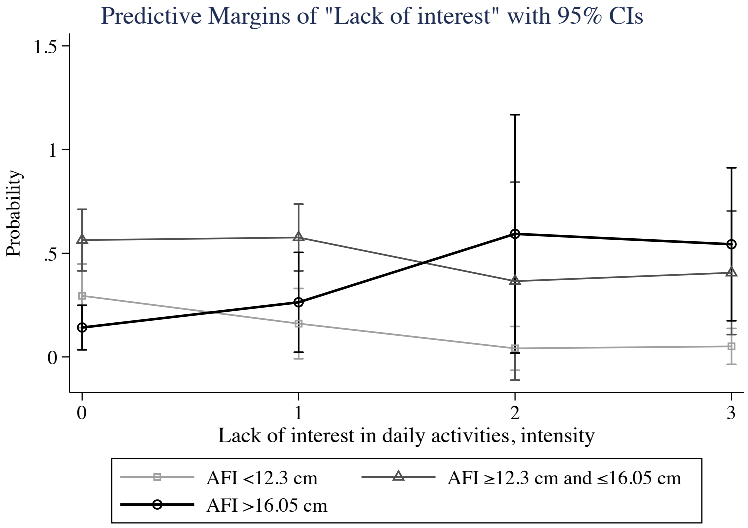

B

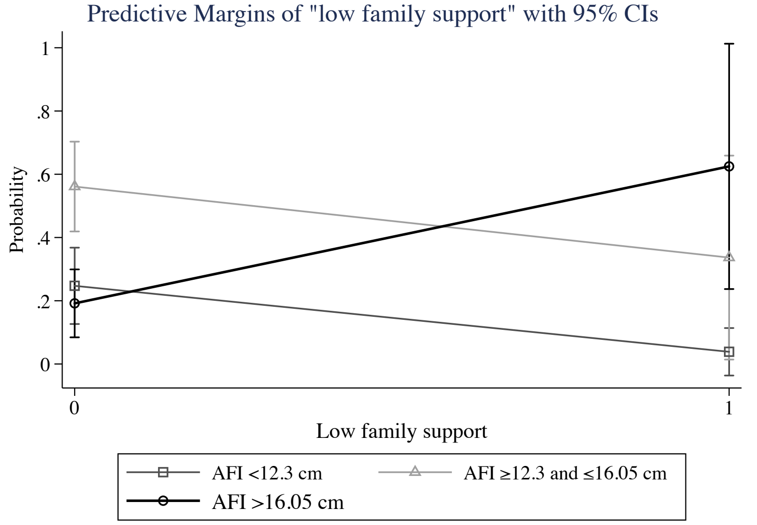

Supplement: Supplementary file 1 [file Data_Sheet_1.PDF]
